# Outcomes of Chronic Anterior Shoulder Dislocation Treatment: A Systematic Review

**DOI:** 10.7759/cureus.100831

**Published:** 2026-01-05

**Authors:** Cry Mabaso, Maradona Mashigo, Collen Nkosi

**Affiliations:** 1 Orthopaedic Surgery, University of the Witwatersrand, Johannesburg, ZAF

**Keywords:** anterior, arthroplasty, bristow-latarjet procedure, chronic, dislocation, instability

## Abstract

Chronic anterior shoulder dislocation, defined as a glenohumeral dislocation that remains unreduced for at least three weeks, is a rare yet debilitating condition marked by progressive soft tissue contractures, bone loss, and altered joint stability. This review aims to provide a systematic review of the literature and a proposed treatment algorithm for this injury pattern.

This systematic review was conducted according to the Preferred Reporting Items for Systematic Reviews and Meta-Analyses (PRISMA) criteria. We performed a search of PubMed, Excerpta Medica database (Embase), Google Scholar, and Cochrane Database of Systematic Reviews electronic databases to retrieve articles published from January 1974 to December 2024. We included studies of patients treated both nonoperatively and operatively for chronic or neglected anterior shoulder dislocations, defined as lasting three weeks or longer. The initial search result provided 427 studies to be assessed. Seventeen papers were included in this systematic review following meticulous screening. There were 242 patients, consisting of 100 males and 142 females, managed for chronic anterior shoulder dislocation across all of the studies, with a weighted mean age of 58.4 ± 13 years. This study has three therapy groups: an untreated cohort, a closed reduction percutaneous pinning cohort, and an operative cohort, by way of open reduction and bone block procedures, soft tissue procedures, or arthroplasty. The untreated group and the closed reduction percutaneous pinning group had lower complication rates but worse functional results compared to the operated group of patients.

Management of chronic anterior shoulder dislocations has been overlooked as compared to management of acute shoulder instability, with no definitive guidance for the orthopedic community to aid the aging population affected by this devastating condition. Despite the procedures outlined in the literature, none have demonstrated superiority in terms of positive outcomes. There is a deficiency of Level 1 studies regarding this condition.

## Introduction and background

Chronic anterior shoulder dislocation, defined as a glenohumeral dislocation that remains unreduced for at least three weeks, is a rare yet debilitating condition marked by progressive soft tissue contractures, bone loss, and altered joint stability [1,2]. The global incidence is uncertain due to underdiagnosis and delayed presentation, particularly in resource-limited environments where reliance on traditional therapies is common [1,3]. From the available literature, chronic anterior shoulder dislocation makes up roughly 10%-15% of missed anterior dislocations and predominantly affects elderly patients following low-energy trauma or younger patients with seizures and alcoholism [4, 5, 6].

Different treatment modalities of chronic anterior shoulder dislocation have been outlined in the literature. They include conservative management, which mainly consists of closed reduction with or without percutaneous pinning, and operative management, which can be divided into open reduction and bone block procedures such as the Latarjet procedure, soft tissue procedures, and arthroplasty. Initial conservative management may be considered in unwilling or frail patients. In Theophile et al.'s Cameroonian cohort, 79.4% of patients achieved closed reduction with good functional recovery, though outcomes remain the exception [7]. Most studies report poor Rowe scores compared to their surgically treated counterparts [1]. Sahu et al.’s systematic review confirmed similarly poor outcomes with nonsurgical treatment, highlighting high rates of persistent pain and dysfunction [8]. Surgical options vary and include open reduction, Latarjet, Bankart repair, remplissage, bone grafting, and arthroplasty, with no universal consensus [2, 3, 9]. Head-preserving procedures remain preferred for younger patients to avoid early arthroplasty, despite the risks of re-subluxation and arthrosis [8].

Functional improvements have been consistently reported; Rouhani et al. demonstrated an average Rowe score of 86/100 after open reduction and Bankart repair [2]. Goga and Yang noted satisfactory outcomes using the Latarjet and coracoid transfer techniques with low complication rates [4, 5]. Benabdallah et al., in a large Moroccan cohort, reinforced open repair’s superiority over neglect with a mean Rowe score of 74 in surgically treated patients [3]. This review aims to provide a systematic review of the literature and a proposed treatment algorithm for this injury pattern. We hypothesized that clinical outcomes would be similar for patients who underwent surgical intervention compared to those managed conservatively in chronic anterior shoulder dislocation.

## Review

Methods

Search Strategy 

A systematic review of the literature on chronic shoulder dislocation was conducted using PubMed, the Excerpta Medica database (Embase), Google Scholar, and the Cochrane Database of Systematic Reviews according to the guidelines presented in the Preferred Reporting Items for Systematic Reviews and Meta-Analyses (PRISMA) statement. The review performed included all articles published from 1974 to December 2024 with chronic anterior shoulder dislocations treated both non-operatively and operatively in the English literature. Furthermore, the reference lists of articles that met the inclusion criteria were manually reviewed to identify additional relevant studies.

Search Criteria 

We included studies of patients treated both nonoperatively and operatively for chronic or neglected anterior shoulder dislocations, defined as lasting three weeks or longer. Studies that reported follow-up outcomes were included. We excluded studies with acute shoulder instability, posterior chronic or neglected shoulder dislocations, and systematic review studies. Search terms included (chronic or unreduced) AND anterior AND (shoulder OR glenohumeral) AND (dislocation or instability). 

Data Extraction

The data extracted from the studies that were selected included the primary author, year of publication, study design, patient demographics, treatment modality, duration of follow-up, complications, outcome measures and their findings, as well as reported P values. All data were documented in a pre-formatted Microsoft Excel (Microsoft Corp., Redmond, WA, USA) spreadsheet for subsequent analysis. The search strategies were executed without filters to minimise the number of missed results. The weighted mean was used to get the average for all included studies. 

Quality Assessment 

The quality of the included studies was assessed using the modified Newcastle-Ottawa scale (mNOS, Table 1), which consists of nine items that evaluate subject selection, cohort comparability, and outcomes to determine both internal and external validity. Scores of 10, out of a maximum of 12, were deemed to reflect high methodological quality, while scores ranging from 7 to 9 were categorised as intermediate quality, and scores below 7 were indicative of low quality. Independent quality assessments of the included studies were conducted by two reviewers, as outlined in the Cochrane Handbook for Systematic Reviews of Interventions. Discrepancies were addressed through discussion, and when needed, a senior author was consulted [10,11].

**Table 1:** Study quality appraisal using the modified Newcastle Ottawa Scale Source: [10]

Selection	Decision rule
1	Was the cohort of the undergoing chronic anterior shoulder management representative? (somewhat representative=1, truly representative= 2)
2	Was the selection of the patient cohort drawn from the same community? (yes=1)
3	Was patient demographic and clinical information adequately verified? (secure record= 2, structured interview or self-report= 1)
4	Demonstration that the outcome of interest was not present at the start of the study? (yes=1)
Comparability	
1	Did the study report age, sex, and comorbid conditions? (yes=1)
2	Did the study differentiate the results according to treatment type? (yes= 1)
Outcomes	
1	Was the assessment of outcome/s of interest clearly defined? (yes=1)
2	Was the follow-up long enough for outcomes to occur? (>1 yr=1)
3	Adequacy of follow-up of cohorts (100% of patients completed follow-up 2, >90% of patients completed follow-up 1)

Statistical Analysis 

The data were collected, entered in Microsoft Excel, and analysed using the STATA software, version 17 (Stata Corp, College Station, TX, USA). Descriptive statistics were used to summarise the results. Categorical variables were presented as frequencies and percentages. Due to significant heterogeneity between studies, variable methods, and a lack of directly comparable data, it was not possible to conduct a meta-analysis or statistical evaluation.

Ethical Considerations

This study solely examined existing published data, thus negating the need for approval from a designated ethics committee. This systematic review was conducted in adherence to internationally recognised ethical principles, specifically concerning confidentiality and scientific integrity.

Artificial Intelligence

This research did not utilise artificial intelligence, nor was it involved in the communication of the results. All data analyses and interpretations were carried out manually by the researchers participating in the study.

Results

Initial Search Results

**Figure 1:**
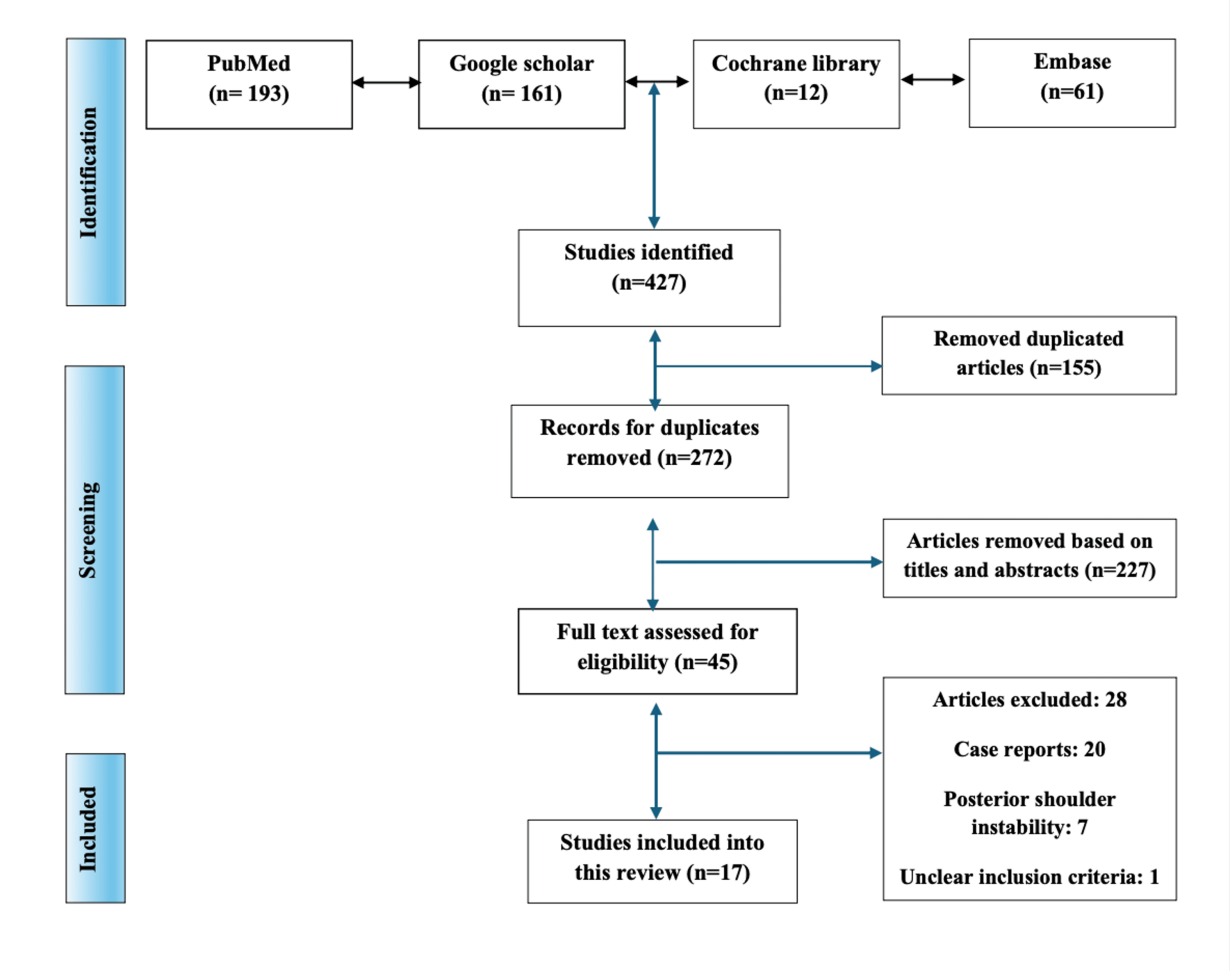
A PRISMA flowchart outlining the study selection process PRISMA: Preferred Reporting Items for Systematic Reviews and Meta-Analyses; Embase: Excerpta Medica database

A search of the database yielded 427 articles; following manual de-duplication, 272 articles were retained. Following the screening of titles and abstracts, a total of 45 articles were selected for full-text analysis. The citation search did not produce any further studies. Following this process, 17 articles satisfied the inclusion criteria and were included in the data extraction process (Figure 1) [1-4,12,13,14-24]. 

Article Quality Results

Of the 17 included articles, all were observational studies (14 retrospective [1-4,12-17,19-23] and three prospective [14,18,24]). The critical appraisal of study quality identified 12 studies as high quality [2,4,13-6,18,19-22,24] and four as intermediate quality [1,3,17,23]. One study was found to be of low quality [12]. The average mNOS score across all studies was 9.65, with a range of 7 to 12.

Study Characteristics and Demographics

A total of 17 studies were included in this systematic review, comprising eight retrospective cohort studies [4, 3, 12,13,17,19,21,22] and nine case series [1, 2, 14-6,18,20,23,24]. A total of 242 patients satisfied the inclusion criteria and were included in this systematic review [1-4,12-24] (Table 2). All 17 studies included sex, indicating that 100 (41.3%) patients were male and 142 (58.7%) patients were female [1-4,12-24]. Eleven studies reported the side involved, with the right side affected in 110 cases and the left side in 43 cases [1-4,12,15,16,18,20,22,24]. Out of the 242 cases, three patients had bilateral shoulder involvement [4, 18], resulting in a total of 245 shoulders and 242 cases with unilateral involvement [1-3,12-17,19-24]. Hand dominance was reported in seven studies, with the dominant hand involved in 74 cases and the non-dominant hand involved in 25 patients [2,4, 12,14,19,21,24].

**Table 2:** Summary of demographic characteristics of patients across the included studies

Authors	Year	Study type	Study design	Mean age (years)	Population size
Flatow [12]	1993	Research article	Retrospective	67	17
Matsoukis [13]	2006	Research article	Retrospective	69.3	11
Raiss [14]	2009	Case series	Prospective	67	10
Rouhani [2]	2010	Case series	Retrospective	42.1	8
Akinci [15]	2010	Case series	Retrospective	39,7	10
Abdelhady [16]	2010	Case series	Retrospective	27.3	4
Werner [17]	2014	Research article	Retrospective	71	21
Babalola [19]	2015	Case series	Retrospective	42	9
Abdelhady [18]	2015	Case series	Prospective	26.5	5
Li [19]	2016	Research article	Retrospective	55.1	25
Van Tongel [20]	2016	Case series	Retrospective	73	6
Statz [21]	2017	Research article	Retrospective	62	19
Su [22]	2017	Research article	Retrospective	60	13
Benabdallah [3]	2018	Research article	Retrospective	44	53
Frias [23]	2018	Case series	Retrospective	69.5	6
Yang [4]	2020	Research article	Retrospective	60.94	18
Rai [24]	2022	Case series	Prospective	47.1	7

Fifteen studies involving 213 cases reported the mechanism of injury [1-4,13-18,20,22,23,24]; two studies did not provide this information [19,21], and one study mentioned it for some patients [12]. The majority of cases, 118, resulted from falls [1-4,12,15,17,18,22,24], while 69 cases were attributed to unspecified trauma [13,14,20,23]. Additionally, 10 cases were injured due to seizures [12,16,18], eight cases were the result of motor vehicle accidents [1, 3,4,15,24], and three cases were classified as undetermined [3,4,12]. Two cases involved injuries sustained during sports [3, 15], one case occurred at work [3], one case involved a wringer injury [4], and one case was due to forced abduction and hyper-external rotation [15].

Management 

Close reduction treatment: Twenty patients had closed reduction treatment [3,22], and one patient was lost to follow-up [3]. Fourteen cases exhibited stability post-reduction [3, 22], three were unstable following reduction [3], and three had closed reduction with pinning [3].

Non-operative treatment: Thirty-one patients underwent non-operative management [1,3,12], while three cases were lost to follow-up [3]. Seven cases were managed non-operatively due to the patients being unfit for surgery [12], while the remaining cases did not mention indications for intervention [1,3]. Seven cases underwent strengthening exercise rehabilitation [12].

Operative management: A total of 195 shoulders were operated on, including three patients with bilateral shoulder injuries [1-4,12-24]. Fourteen studies reported indications for surgery [1-4, 12,13,14,15,17-19,21,23,24], while two studies did not specify the indications [16,20]. The fourteen studies identified pain and limitation as indications for surgery [1-4, 12-15,17-19,23,24].

Out of the 195 shoulders that underwent surgical treatment, 82 (42.3%) were subjected to arthroplasty [12,13,14,17,20,21,23], comprising 11 hemiarthroplasties [13,21], 19 total shoulder arthroplasties [12,13,21], 10 resurfacing shoulder arthroplasties [14], and 42 reverse shoulder arthroplasties [17,20,21,23]. Open reduction with soft tissue repair or bony block procedures were performed on 107 shoulders, which included the Boytchev, Latarjet, Bristow, and Putt procedures [1-4, 12,15,16,18-20,24]. Additionally, only five shoulders had a combined Latarjet surgery and hemiarthroplasty [19].

Follow-Up 

All 17 studies reported on the duration of follow-ups [1-4,12-24 ]. The average follow-up period was 31.0 months, with a range of eight to 98.4 months [1-4,12-24].

Range of Motion 

Thirteen studies provided information on the clinical range of motion [2,4,12,17,18,19,21,23,24], while four studies did not address this aspect (Table 3) [1,3,20,22]. Six of the studies involved patients who underwent arthroplasty [12,13,14,17,21,23], while the other seven studies included patients who had open reduction with bony block, soft tissue repair, or concomitant hemiarthroplasty [2,4, 15,16,18,19,24].

**Table 3:** Pre- and postoperative range of motion

Authors	Forward flexion (degrees)		External rotation (degrees)		Internal rotation (degrees)		Abduction (degrees)	
	Preoperative	Postoperative	Preoperative	Postoperative	Preoperative	Postoperative	Preoperative	Postoperative
Flatow [12]	-	147	-	69	-	-	-	-
Matsoukis [13]			13.2	25.5	-	-	-	-
Raiss [14]	45.1	134	7.7	34	-	-	30.8	126.5
Rouhani [2]	-	122	-	22.5	-	T12	-	-
Akinci [15]	44	88.5	4.2	11.5	38	91	6	13.5
Abdelhady [16]	-	135	-	27.5	-	60	-	125
Werner [17]	35	128	2.4	8.4	-	-	25	113
Abdelhady [18]	-	-	-	46	-	-	-	-
Li [19]	-	97.6	-	21.6	-	L3	-	-
Statz [21]	51	94	1	34	Iliac crest	Sacroiliac joint	-	-
Frias [23]	-	105	-	18	-	L3	-	-
Yang [4]	67.5	115	9.4	30	-	L2	-	110
Rai [24]	-	-	-	-	-	-	37.7	108

Functional Outcomes

A mean postoperative subjective value of 43 [21] and an average score of 5.7 on the Simple Shoulder Test were reported in a single study involving shoulders that underwent arthroplasty [21] exclusively, including reverse total shoulder arthroplasty, total shoulder arthroplasty, and hemiarthroplasty for locked anterior shoulders.

Two studies examined the postoperative American Shoulder and Elbow Surgeons (ASES) score. One study focused on an arthroplasty cohort, reporting a mean postoperative score of 65 [21]. The second study involved a cohort undergoing the Bristow-Latarjet procedure, with a mean preoperative score of 39.1 and a mean postoperative score of 83.4 [4].

Three research reports on the University of California Los Angeles (UCLA) Score; two of the studies had patients that underwent the Bristow-Latarjet procedure [4,24], with a mean preoperative UCLA score of 9.3 (range: 8.4-10.2). and postoperative UCLA with a mean of 23.5 (ranging from 22.5 to 24.5). A single cohort was comprised of patients who underwent the Bristow-Latarjet procedure, while another group received simultaneous humeral head replacement, with a combined mean postoperative UCLA Score of 19.6 [19].

Four studies reported on the postoperative visual analogue scale (VAS) [4,19,21,24]. Two studies treated their cohort with the Bristow-Latarjet procedure, with a preoperative VAS mean of 6.25 and a postoperative mean of 1.85 [4,24]. A study focused exclusively on arthroplasty cases reported a preoperative VAS score of 4.7/5 and a mean postoperative score of 2.2/5 [21]. The final study among the four involving patients who underwent the Bristow-Latarjet procedure, including some cases with simultaneous humeral head replacement, reported a VAS mean postoperative score of 2.3 [19].

The Rowe score was documented in four studies [1,2,18,24]. One study had a cohort that was treated with open reduction and simultaneous Bankart lesion repair; the mean postoperative Rowe score was 86.3 [2]. The second study included two groups of patients [1]: the operative group, which underwent open reduction and capsulolabral repair using Kirschner wire or Steinmann pin fixation, and the non-operated group. The mean Rowe score for the operative group was 44.6, while the non-operated group had a mean score of 24. The last two studies included patients who underwent the Bristow-Latarjet procedure. One study reported both preoperative and postoperative Rowe scores of 28.8 [24] and 76.6 [24], respectively, while the other study reported only the postoperative Rowe score of 73.3 [1].

Nine studies reported on the Constant-Murley (CM) score in five patients who underwent arthroplasty [13,14,17,20,23], followed by three studies involving open reduction with bony block and/or soft tissue repair and/or hemiarthroplasty [16,18,19], as well as one study on closed reduction (Table 4) [22].

**Table 4:** Constant-Murley score

	Preoperative (points)	Postoperative (points)	Pre-reduction (points)	Post reduction (points)
Arthroplasty (mean) [13,14,17,20,23]	19.95	60.05	-	-
Arthroplasty (median) [13,14,17,20,23]	-	65	-	-
Open reduction and bony block or/ and soft tissue repair or/and hemi arthroplasty (mean) [16,18,19]	17	70.5	-	-
Closed reduction (mean) [22]	-	-	34.35	60.94

Complications 

Of the 195 shoulders that underwent surgery [1-4,12-24], 56 (28.9%) shoulders had complications [1-4,12-15,17-19,21], and seven shoulders (2.9%) underwent reoperation [3,21,22,24] (Table 5).

**Table 5:** Summary of postoperative complications

Authors	Revision	Re-dislocation/ instability	Glenoid loosening	Glenoid erosion	Pin track infection	Post-traumatic osteoarthritis	Nerve injury	Surgical site infection	Persistent pain	Positive apprehension	Non-union	Lateral graft	Fracture	Heterotopic ossification
Flatow [12]	1	1	-	-	-	-	-	-	-	-	-	-	-	-
Matsoukis [13]	2	4	1	-	-	-	-	-	-	-	-	-	-	-
Raiss [14]	2	1	-	-	-	-	-	-	1	-	-	-	-	-
Rouhani [2]	-	2	-	-	-	1	-	-	-	-	-	-	-	-
Akinci [15]	-	-	-	-	2	-	-	-	1	-	-	-	-	-
Abdelhady [16]	-	-	-	-	-	-	-	-	-	-	-	-	-	-
Werner [17]	2	-	2	-	-	-	-	-	-	-	-	-	-	-
Babalola [1]	-	1	-	-	-	-	-	-	-	-	-	-	-	-
Abdelhady [18]	-	-	-	-	-	-	-	-	-	1	-	-	-	-
Li [19]	-	12	-	-	-	15	-	-	-	-	1	2	-	-
Van Tongel [20]	-	-	-	-	-	-	-	-	-	-	-	-	-	-
Statz [21]	-	6	1	2	-	0	-	-	-	-	-	-	3	1
SU [22]	-	-	-	-	-	-	-	-	-	-	-	-	-	-
Benabdallah [3]	-	1	-	-	--	-	-	2	-	-	-	-	-	-
Frias [23]	-	-	-	-	-	-	-	-	-	-	-	-	-	-
Yang [4]	-	1	-	-	-	-	1	-	-	-	-	-	-	-
Rai [24]	-	-	-	-	-	-	2	-	-	-	-	-	-	-

Discussion

All studies included in this systematic review were classified as level IV studies [1-4,12-24], consisting of nine case series [1,2,14,15,16,18,20,23,24] and eight retrospective full articles [3,4,12,13,17,19,21,22], which demonstrate that this type of orthopaedic condition is rare and seldom reported by researchers. Sixteen of the 17 studies that were included were published between 2006 and 2022 [1-4, 13-24]. This review offers some optimism that more orthopaedic surgeons are embracing the opportunity to operate on these shoulders and are starting to share their outcomes.

In this systematic review, 17 studies were analysed [1-4,12-24], revealing that the mean age of shoulders undergoing arthroplasty was 68.1 years (range: 62-73 years) based on seven studies [12,13,14,17,20,21,23]. Among patients who underwent open reduction with soft tissue repair and/or bony block and/or concomitant hemiarthroplasty, the mean age was 42.9 years (range: 26.5-60.94 years) across nine studies [1- 4, 15,16,18,19,24]. For the cohort with closed reduction, the mean age was 53.7 years (range: 47.4-60 years) from two studies [3,22]. Lastly, the cohort receiving non-operative treatment had a mean age of 48.05 years (range: 46.4-49.75 years) based on two studies [1,3]. Three of the 17 studies included patients who had mixed treatment per cohort [1,12,23].

Su et al. [22] reported on patients who were treated with closed reduction, with a mean presentation time of four weeks and a mean age of 60 years. The post-reduction CM score improved significantly; they concluded that this method is feasible for selected patients using the Hippocratic approach. This systematic review included only two studies involving patients treated with closed reduction: Su et al. [22] and Benabdallah et al. [3].

Benabdallah et al. [3] reported on 20 patients who received non-operative therapy, indicated by either refusal or abstinence from intervention. In the follow-up of 17 patients, 14 showed good to excellent outcomes, while three revealed poor to fair results. Babalola et al. [1] observed poor functional results with non-operative treatment, as patients had a Rowe score of 24. Flatow et al. [12] reported that non-operative treatment leads to considerable functional impairments.

A total of 111 shoulders, which represented the largest group in the study, underwent open reduction and/or soft tissue repair, bony block, or simultaneous hemiarthroplasty [1-4,15,16,18,19,24]. Yang et al. [24] reported on shoulders that underwent the Bristow-Latarjet procedure, with a mean waiting period of 73.3 days prior to surgery. The mean age of the patients was 60.94 years, and the mean follow-up duration was 15.2 months. Only one of the 20 shoulders experienced postoperative subluxation. The authors concluded that the Bristow-Latarjet procedure yields acceptable clinical outcomes. Rouhani et al. [2] reported on a patient who underwent open reduction and simultaneous Bankart lesion repair after a 10-week waiting period. The mean age was 42 years, and Rowe and Zarin's score was 86 points at a mean follow-up of 12 months. The overall complication rate was 37.5%.

Shoulders that underwent arthroplasty were 82 [12,13,14,17,20,21,23]. Raiss et al. [14] reported cases requiring humeral head resurfacing due to fixed anterior glenohumeral dislocation. They noted favourable clinical outcomes and a moderate rate of complications in the short term, with 20% of the cases necessitating reoperation. Statz et al. [22] reported cases involving arthroplasty for locked anterior shoulder dislocation. Reoperation needed to be performed on 15.8% of the shoulders, with 10.5% undergoing total shoulder arthroplasty due to early redislocation and 5.3% hemiarthroplasty for late, painful glenoid arthrosis. At the final follow-up, 21.1% of additional shoulders (10.5% anatomic shoulder arthroplasty and 10.5% hemiarthroplasty) exhibited instability. The study concluded that reverse shoulder arthroplasty offers superior pain relief and enhanced range of motion when compared to total shoulder arthroplasty and hemiarthroplasty. Reverse shoulder arthroplasty is likely indicated in these challenging circumstances.

Shoulders that underwent arthroplasty treatment [12,13,14,17,20,21,23] exhibit a better range of motion compared to those that had open reduction with soft tissue repair, or bony block procedures, or simultaneous hemiarthroplasty interventions [1-4,15,16,18,19,24]. The functional outcomes were more favourable in shoulders that underwent open reduction with soft tissue repair or bone block compared to those that received arthroplasty.

Preoperative and postoperative outcome measures for shoulders that underwent surgery showed improvement [1,2,4,12-24]. These include the CM score, Rowe and Zarins score, VAS, American Shoulder and Elbow Surgeons Shoulder score, UCLA shoulder score, Simple Shoulder Test score, and subjective shoulder value [1,2,4,12-24].

The overall complication rate was 22.9% (56 of 245) [1-4,12-15,17-19, 21], with arthroplasty contributing 28.1% (23 of 82 shoulders) [13,14,17,21] and open reduction with soft tissue repair or bony block accounting for 29.5% (33 of 112 shoulders) [1,2,4, 12,15,18,19,23,24]. The complication rates are similar, making it difficult for the surgeon to determine the best type of operation.

## Conclusions

Our study indicates that favourable clinical and functional outcomes are attainable through early closed reduction and surgical intervention in patients with chronic anterior shoulder dislocations. Our observation from this data is that arthroplasty is predominantly favoured for patients aged over 61 years, while open reduction combined with soft tissue procedures or bony block is preferred for those younger than 61 years. No implant has been designed to treat this situation; bony blocks and soft tissue are intended for instability, while arthroplasty implants are not formulated to rectify this condition. We recommend reverse shoulder arthroplasty for patients over 60 years of age, as it demonstrates superior clinical outcomes compared to anatomic shoulder arthroplasty, hemiarthroplasty, and the Bristow-Latarjet procedure in patients under 60 years, owing to improved functional results. Close reduction should always be attempted when feasible by the orthopaedic surgeon.
